# The proposed need for integrated maternal and child oral health policy: A case of South Africa

**DOI:** 10.3389/froh.2022.1023268

**Published:** 2022-12-02

**Authors:** Khabiso Ramphoma, Nashna Rampersad, Nuerisha Singh, Ntsakisi Mukhari-Baloyi, Sudeshni Naidoo

**Affiliations:** ^1^ Department of Community Oral Health, Faculty of Dentistry, University of the Western Cape, Cape Town, South Africa; ^2^ Department of Community Dentistry, School of Oral Health Sciences, Sefako Makgatho Health Sciences University, Pretoria, South Africa

**Keywords:** childhood dental caries, maternal and child health, oral health policy, integrated primary health care services, oral health

## Abstract

The high oral disease burden among children in South Africa, specifically early childhood caries, has received scant attention despite the fact that it is a public health concern that negatively impacts the overall well-being and quality of life of the child. While South Africa has a number of well documented policies that focus on oral health in general and maternal and prenatal health, none specifically addresses the oral health of children under the age of six years. The integration of oral health in maternal and child health care in South Africa could lead to an improved oral health quality of life and better oral health outcomes for mothers and children to address the high prevalence of childhood caries and unmet treatment needs for this population. While the integration of oral health care into primary healthcare is recognised as crucial and affordable, it however continues to be neglected. In South Africa, oral health disparities are attributed to the unequal distribution of oral health services, and policies that govern oral, maternal and child health seem to work in parallel with one another. Integrating oral health into interventions for primary health care delivery is a cost-effective way to improve the health of disadvantaged groups. Considering that maternal oral health predicts children's oral health and primary health care teams regularly see under-6-year-olds, this primary care setting is ideal for integration of these services. Despite growing interest in an integrated oral health and primary care system, there is little literature on oral health integration models. Improving the oral health of vulnerable populations requires integrating oral health into primary care and implementing interdisciplinary public health programs. The development of an Integrated Maternal and Child Oral Health policy would play a critical role in advancing integration; however, such a policy should be designed with both implementation and translation in mind for it to be successfully followed through. Such a policy should be comprehensive and contextual, aimed at increasing access to oral health services for women and children and reduce the oral disease burden. This paper proposes and describes the possible content and objectives of such a policy that will enhance effective leadership and accountability and strengthen health system delivery platforms for quality maternal and child oral health services along the continuum of healthcare. Furthermore, it will illustrate the importance of a policy that aims to promote coordinated, relevant, trans-multi-disciplinary and inter-sectoral community engagement to improve pregnancy and oral health outcomes, and importantly, establish a sustainable and contextual surveillance system for maternal and child oral health.

## Introduction

Policy development is an important public health function and is required for the promotion of population health and its improvements (1). Health policies are instrumental in providing a direction towards which governments aspire to be, as well as give an indication of the resources required to achieve the intended aspirations (2). As mandated by the *National Health Act of 2003*, the South African Government's Department of Health, is responsible for establishing policies and laws pertaining to the rights and responsibilities of its citizens, as well as the delivery of services in the public health system. National policies are based on the political priorities of a government that aim to inform the public of the government's vision and goals and respond to the country's societal challenges, rule of law and democratic values (3).

The underlying philosophy of South Africa's National Policy on Oral Health (NPOH) was based on the primary health care approach, with the intention of ensuring optimum oral health by preventing dental disease and promoting the oral health of the South African population (4). However, in South Africa, oral health services delivered through the public health system are inequitably distributed (5). Furthermore, limited access to oral health care exacerbates the burden of oral diseases (6), and dental caries is the most common oral disease in South Africa7. Specifically in the child population, it has been estimated that approximately 60% of children are affected by early childhood caries by the age of six years (7).

Several interventions have been advocated to curb the burden of childhood dental caries in the under six population with very little progress (8), however, in resource limited settings as in South Africa, more creative, innovative and efficient interventions should be sought. Maternal care-related attitudes, motivation, and beliefs significantly influence children's oral hygiene practices and a correlation between maternal oral health and behaviours and childhood caries has been reported (9). Additionally, the World Health Organization (WHO), advocated that an integrated approach to public health problems, such as dental caries in children under six-years of age, can make significant progress on a population level (10). As such, the integration of oral health care into primary health care has been recognised as being key to the provision of affordable and accessible care, especially since oral health is still neglected in many developing countries (11).

On the premise that both maternal and child health care, as well as oral health care in South Africa is based on a primary health care approach, the integration of these services could potentially lead to better oral health outcomes for both mothers and children. However, in order to effectively integrate these two approaches and respond to the societal challenges these populations face, the process of developing an Integrated Maternal and Child Oral Health (IMCOH) policy is required. The purpose of the present review is to propose the development of an IMCOH policy, specifically for the South African context.

## Prevalence and impact of dental caries in children

### Globally

Oral diseases are a major public health concern, affecting over 3.5 billion people worldwide (12). More specifically, dental caries contributes significantly to the global burden of oral diseases. It is estimated that over half a billion children worldwide have untreated caries of the primary dentition, which can have a significant impact on their quality of life (13).

Early childhood caries (ECC) is defined as the presence of at least one decayed (cavitated or non- cavitated), filled, or missing (due to caries) teeth in children under the age of six (14). The consequences of ECC on the oral health, general health, and well-being of children can last a lifetime (15). When left untreated, dental caries can impair a child's ability to function, limit their participation in daily activities, and even have an effect on their development (16). Even though considerable progress has been made, dental caries in children under six remains a worldwide problem (17).

In the global context, the circumstances and prevalence for dental caries in children varies (15). In the United Kingdom in 2015, nearly 23% of children aged 5 years were found to have ECC. In Australia, the 2012–2014 National Child Oral Health Study reported that 34% of children aged between 5 and 6 years had caries in their primary teeth and 26% had untreated decay in their primary dentition (15). The situation is dire for developing countries. By the age of five, more than half of Brazilian children have dental caries, with 80% going untreated (15). China's most recent national oral health survey found more than 70% of Chinese children aged 5 had dental caries (15).

### South Africa

Similar to many developing countries, South Africa has a significant burden of ECC. This has put great burden on the public health system as high numbers of children require dental treatment for ECC under general anaesthesia (18). In addition, there are high levels of unmet treatment needs among South African children8. The most recent oral health survey in South Africa (1999–2002), focused on children only, found that 60.3% of children under the age of 6 years had caries, and 80% of this group had untreated caries (19). As there are no health information systems that monitor or carry out sentinel surveillance of oral health diseases, the overall current oral health status of children in South Africa is unknown (2). However, recent studies have reported that high ECC prevalence and high unmet treatment needs persists among South African children. Kimmie-Dhansay et al., reported that the lifetime prevalence of ECC for South African children under 71 months was 44.5% (20). Specifically, in 4- and 5-year-olds, the overall prevalence of ECC increased from 57% between 1975 and 1979, to almost 62% between 2010 and 2014.

The prevalence of ECC in rural areas was higher than the urban areas. Mohamed and Barnes (21) found that 67.5% of children (between 6 months and 6 years old) from low socioeconomic communities in the Western Cape Province had evidence of dental caries that necessitated some form of dental treatment. Furthermore, the unmet treatment need of 4- to 5-year- old children in the Western Cape increased since the previous National Children's Oral Health Survey (1999–2002) and shows that the problems persist and have not been addressed (21). Similarly, in the Province of Kwa-Zulu Natal, Reddy & Singh (2015), reported that more than 70% of 6-year olds were affected by dental caries, with nearly all (94%) requiring dental treatment (22).

Several risk factors have been proposed as contributing to the increased prevalence of ECC. These include oral health behaviors, socioeconomic status, and other family-level factors such as education levels, ethnicity, health attitudes and family size, among others (23). Early childhood caries have a significant global impact, and requires prompt intervention to improve children's oral health (24). The availability of oral health care is one of the structural risk factors for ECC that can be mitigated by government policy (15).

## Public health services in South Africa

Due to historical racial prejudice, poverty, unemployment, a lack of access to health care, and poor educational quality, South Africa has the world's largest economic disparity between its citizens (25). The majority of South Africans are dependent on the public health system and social services to meet the demands of the population. Although the delivery of quality and equitable health care, including oral health care, is a constitutional obligation in South Africa (26), many disadvantaged communities, that make up the majority of the population, have limited access to healthcare. These disparities of oral health care services in the public health system are exacerbated by a lack of oral health facilities and an adequate oral health workforce (27). Postma, Ayo-Yusuf and van Wyk (2008), highlighted that the majority of ECC studies are descriptive in nature with the main focus on particular geographical areas, hence the lack of priority to address this issue. Additionally, they concluded that the implementation of an integrated primary oral health care strategy would effectively address ECC in South Africa (28).

Access to basic health care has improved in South Africa over the past two decades (29), which is evident in maternal and child mortality rates, among other indicators. Neonatal mortality, infant mortality and the mortality of children under five-years of age has been markedly reduced. Infant deaths significantly decreased from 46 per 1,000 live births in 1995, to 15 per 1,000 live births in 2012 (29). Although child and maternal mortality rates have decreased, they remain disappointingly high when compared to similar countries (29). The availability and affordability of health care services remain significantly constrained – nearly half of the population have inadequate health care that is not readily available and affordable (30). This is the same situation for oral health care (5).

Smit & Osman found that the availability of the basic oral health care package in public dental clinics in the Western Cape Province was poor and disproportionately distributed among rural and urban public health facilities (5). They reported that less than a third of the Province's clinics offered the package, and suggested that marginalized populations serviced by satellite clinics were at an even greater disadvantage with only 15% of these clinics offering the basic package. Furthermore, the study reported that the availability of dentists at public clinics in the entire Province was low (mean number of 7.3 days/month) and the situation was worse at satellite clinics where dentists were present for only 3.2 days per/month (5). It can therefore be anticipated that communities with these demographics face the highest prevalence of oral diseases. Consequently, many South Africans delay dental care and this may have long-term consequences for their physical and mental health (31). These factors place South African children at a higher risk of acquiring oral diseases, such as dental caries.

## Existing health policies in South Africa

South Africa has several policies that support increasing access to health and creating supportive environments. Although the policies focus on the primary goal of achieving health for all, there are separate policies for some vulnerable groups, such as for women and children.

The National Policy for Oral Health (NPOH) in South Africa was revised after 1990 from the National Dental Policy that was approved by Cabinet in 1975. This process was necessitated by the National Policy for Health Act, 1990 (Act 116 of 1990). On reviewing South Africa's NPOH, Mukhari-Baloyi et al., found that while the policy's goals were clear, it did not provide direction on how to achieve them (2). As disease burdens and demand vary by province, a preventive method that is effective in one community may not be suited for another. Consequently, the policy was deemed uninformative. However, it permitted the provinces and districts to implement policies tailored to their own populations (2). Additionally, they reported that the South African NPOH was outdated (2) and although it defined the oral disease burden affecting the population, it failed to consider comorbidities, lifestyle choices and the determinants of health, many of which predispose individuals to oral diseases (2), and are crucial components of the PHC strategy based on the common risk factor approach.

The NPOH had outlined targeted treatment goals for children in the 6-year-old cohort, suggesting that at least half of them are caries-free by the year 2000.The policy, on the other hand, failed to consider oral health goals for children under the age of six. Although the treatment objectives were not specified, they suggested a focus on preventive measures to meet the stated benchmarks for oral health status (2). Mothers and adolescents would be included by default in the targeted age cohorts above the age of six; however, the objectives as they currently stand cannot be used until the national survey data is updated.

Furthermore, the NPOH does not promote an interdisciplinary approach to health care and does not specify any monitoring and evaluation strategies, such as sentinel surveillance (2). There has been little effort to collect data from public oral health facilities for reporting purposes. This inability to compile national data could be attributed to a lack of consistency in dental records across the country. As a result, determining the effectiveness of the NPOH since its inception has been extremely difficult.

As a child health-focused public health intervention, the Departments of Health and Basic Education established the Integrated School Health Programme (ISHP) that sought to provide school health care services to all students at the primary and secondary school levels. The programme aimed to improve health-promoting behaviors through increased knowledge and awareness, as well as to facilitate the early detection and treatment of health-related learning impediments. Although it has been recognised that oral health screening is an integral component of the ISHP (32), the policy excludes children under the age of six years thereby neglecting the pre-school and crèche-going population.

The South African National Maternal, Child and Women's Health (MCWH) Policy states that an investment in the health of mothers and young children is an investment in the future of the nation. The strength of a health system is reflected in the health status of children, especially when considering the vulnerability of young children (33). However, especially for implementation at district levels, the MCWH policy has been criticized for a lack of a rational, national planning framework, as well as uncoordinated health authorities, which result in unnecessary and high demand for care on tertiary services (34). As a result, the current MCWH draft policy attempts to mitigate those factors, but it, too, makes no mention of oral health of both the mother and the child as part of comprehensive health (33).

In addition, South Africa has a number of well-developed maternal and child health policies, including the South African Maternal, Perinatal and Neonatal Health Policy 2021 and National Integrated Early Childhood Development Policy 2005, among others (35, 36). Although these policies acknowledge the importance of maternal and child health, they do not explicitly mention oral health as an essential component of maternal health care and child development. The country's high burden of infectious diseases such as HIV and tuberculosis has an impact on budgetary priorities, reducing the availability of funding for oral health (27). This further re-enforces the critical and urgent need for an IMCOH policy.

## Policy strategy and implementation

Globally, medical and dental professionals operate independently, though they both offer health promotion, disease prevention, routine services, and emergency care. Due to the lack of infrastructure, technology, and personnel to connect these separate systems, public health patients must traverse the dental-medical divide on their own, frequently without the health literacy skills or resources necessary. Inequities in access to essential health services and poor health are attributable to independently operating dental and medical care systems, which represent missed opportunities to provide services. Using an evidence-based foundation, government, philanthropic, and professional organisations are now adding support for the integration of dentistry and primary health care (37).

Policies and strategic plans for oral health integration in primary care are rooted in the 1978 WHO Alma-Ata declaration. The first strategic plan was published in 1982, and in 2007 the WHO presented “Oral Health: Action Plan for Promotion and Integrated Disease Prevention” to the World Health Assembly, where 193 out of 194 WHO member countries agreed to implement the WHO Global Oral Health Programme. All of these countries have set general policies for oral health action in the last decade, using guidelines and a common risk factor. Due to worldwide variation in oral health care organizational systems, policies on integrated care vary not only between countries, but also within countries at the national and provincial levels (38).

Primary health care (PHC) revitalisation is critical to South Africa's ongoing health reforms as it serves as a platform for service delivery, the facilitation of health promotion, illness prevention, and a first point of entry into the healthcare sector (39). Additionally, initiatives such as the universal health coverage namely the National Health Insurance (NHI) Bill aim to improve the South Africa's ailing healthcare system by increasing access and reducing inequalities. Furthermore, the WHO, advised that relying solely on oral health professionals to deliver oral health care at all levels, including PHC facilities, is unrealistic (10). Consequently, at a population level, an integrated, inter-professional and inter-sectoral approach can make significant progress for public health problems, such as early childhood caries. Primary health care encompasses a vast array of services, such as oral health care, and a variety of public, private, and non-governmental health care providers. Some health care systems have implemented the integration of oral health into primary care to reduce the burden of oral disease and improve access to oral health care, particularly for disadvantaged and underserved communities (40).

In the United States of America, two frameworks have been developed. The first, an Integration of Oral Health and Primary Care Practice (IOHPCP) Initiative, covers five domains including risk assessment, oral health evaluation, preventive intervention, communication and education and inter- professional collaborative practices. This framework has been adopted by some health care centres for the implementation of their tailored programmes (41).

The second, the Oral Health Delivery Framework, developed in partnership with primary care and dental care clinicians, policy makers and stakeholders from medical, dental, and nursing associations, as well as end-users. The activities proposed in this framework promote proactive coordination between dental and non-dental primary care providers and include: screening patients for oral diseases, identifying particularly high-risk populations, offering fluoride varnish for paediatric patients and high-risk adults, performing patient education, dietary counselling and oral hygiene training, and developing structured documentation and referral processes (42). Since 2015, this framework has been implemented in PHC organizations from public services and private practice to examine its feasibility and sustainability.

Integrating oral health into interventions for primary health care delivery are cost-effective methods to improve the health of poor and disadvantaged population groups (10). This would enable the efficient use of the public health systems existing infrastructure and human resources. Especially in South Africa, oral health promotion needs to be easily adaptable to different provincial contexts within South Africa. Its goal is to educate and support policymakers and administrators, as well as staff in primary health care facilities, schools, and local communities, to reduce the burden of oral diseases, especially among the child population (10).

### Rationale for an integrated maternal and child oral health policy

Globally, children aged under six years of age are seen regularly by primary health care teams for vaccinations and general health checks, often by nurses, midwives, and community health workers, and less often by oral health professionals. An oral health policy that is designed and developed to meet the needs of this group will ensure that oral health equity is realized. This would be consistent with the promotion and protection of mothers', women's, and children's rights to health as a basic human right, as emphasized in international health conventions (36).

It is well known that a mother's oral health status is a strong predictor of her child's oral health status (43). Children born to mothers with high levels of untreated caries were more than three times more likely to have higher levels of caries experience (treated or untreated dental caries) than children born to mothers with no untreated caries (44, 45). Similarly, children of mothers with high levels of tooth loss were more than three times more likely to have higher levels of caries experience than children of mothers with no tooth loss (45).

Parental influences play a major role in the caries prevalence of their children, and it has been reported that poor oral hygiene practices of parents and frequent consumption of sweets increased the likelihood of mother-to-child (neonatal) infection transfer. Family living conditions and perceptions also play a role, whereby changes in the family set-up (from two parents to one) may affect the ability of parents to adequately prioritize and care for the oral health of the child, thereby increasing their risk of dental caries (46).

Due to their reliance on parents and caregivers, children face unique oral health challenges. The Fédération Dentaire Internationale (FDI) policy statement on perinatal and infant oral health, advocated that perinatal and infant oral health care are critical components of early intervention, facilitating behavioral changes that result in good oral health, successful caries prevention, and disease management. Importantly, these efforts should be approached through a collaborative, integrated effort by parents, schools, health ministries, and other stakeholders (47).

The WHO's Implementation Manual entitled “Ending Childhood Dental Caries” stated that for the development of a supportive environment, it is important to integrate childhood dental caries prevention and control within primary care, such as maternal and child health programmes to ensure engagement of non-oral health professionals in oral health care (48). It also encourages the development of a national policy to support new skills and competencies of primary care teams, and to ensure their initial and ongoing childhood dental caries prevention and control training (48). In this way, non-oral health professionals will understand the severity and impact of childhood dental caries as a serious public health problem.

On assessing the integration of oral health within MCH services in Tshwane, Molete & Phakela, found that 38% of caregivers with low educational levels did not value the importance of primary dentition and consequently did not take their children to oral health facilities in the first few years of life (49). Furthermore, primary health care mother-to-child clinics did not prioritize oral health since the nurses were overburdened and lacked adequate oral health knowledge. It was also demonstrated that dental practitioners in general practice do not regard themselves as having to play a role in the prevention of oral disease in pre-school children (49).

Globally there is no evidence of integrated mother and child oral health policies, nor of policies that specifically focus on the under six-year-old population for the prevention of childhood dental caries. In Australia, the 2014–2024 National Oral Health Plan serves as a blueprint for action across different disciplines, sectors and jurisdictions, encouraging integrated health care to ensure that Australian children and adults have adequate oral health. In Japan there is a comprehensive governmental strategy (the 8,020 campaign) to improve oral health. More specifically, this strategy explicitly advocates for oral health care for pregnant mothers and children up to 5-years-old (15). Developing an integrated maternal and child oral health policy with policies specific to women and children would enable the creation of a strategic framework. Such a framework can then be adapted using the existing policy, strategy and plan as a guideline to assist with implementation. The National Health Act, 2003 (Act 61 of 2003), the Children's Act, 2005 (Act 38 of 2005), the National Health Insurance (NHI) Bill, the National Oral Health Policy 2010, and the Skills Development Act, 1998 (Act 97 of 1988) are examples of laws and policies into which an IMCOH and be integrated. The guiding principles of these policies envision a shifting narrative for South Africa's political and social landscape for mothers, children, and women.

### Proposed policy for integrated maternal and child oral health (IMCOH)

Brillas and Lee (2022) conducted an integrative review with the aim to identify major maternal and oral health frameworks. They focused on integrating oral health into perinatal care and proposed that such a framework should consider the contextual factors, intervention levels and enabling factors, with policy development being one of the key interventions. Furthermore, they underlined that policymakers need to examine the manner in which oral health is systematically integrated into existing primary health care initiatives through interprofessional training and collaboration (50). A such, an Integrated Maternal and Child Oral Health (IMCOH) policy would need to be comprehensive, focused and contextual. Its vision should encompass the overarching aim of the policy, which in South Africa, will be to increase access to oral health services for women and children, thus ultimately reducing their burden of oral disease. The mission would be to ensure that antenatal, perinatal and neonatal services include oral health education and promotion, screening and referral where necessary. This mission would align with that of the South African Maternal, Perinatal and Neonatal Health Policy (35).

According to the National MCWH policy draft, policy goals are categorized into three main areas for mothers, children and adolescents (33). These goals would seamlessly translate into the objectives of an IMCOH policy if the relevant health workers receive training to execute the required services. An IMCOH policy would establish a broad framework for providing high-quality, all-inclusive maternal and oral health services and should guide the development and revision of guidelines and standard operating procedures for maternal and child oral health at PHC level in South Africa. As with key strategic objectives of the MPNH and the MCWH policies, an IMCOHP can be structured in line with those of the South African National Department of Health (35), with four key objectives: to enhance effective leadership and accountability; to strengthen health system delivery platforms; promote coordinated, relevant, multi- disciplinary and sectoral community engagement to improve pregnancy and oral health outcomes and finally to establish a sustainable and contextual surveillance system for maternal and child oral health.

**OBJECTIVE 1: Enhance effective leadership and accountability to provide quality, comprehensive, and integrated maternal and child oral health care and treatment services across the healthcare continuum**

Human resource needs, planning, as well as management, is essential for the delivery of quality oral health services and should be reflected in integrated policies, with clear roles and responsibilities at each level of government. Although the number of health personnel in South Africa has increased over the years, the increasing burden of disease continues to stress and constrain the public health system. This could be attributed to an increase in the country's young population, urbanization, lack of quality and equitably distributed clinical services and possibly, the lack of appropriate human resource distribution at the various levels of oral service delivery (7).

Appointing Provincial Oral Health Managers is essential to address the significant oral disease burden and poor state of public oral health services in the country. More specifically, the appointment of Community Dentistry specialists into the various government level structures is recommended to co-ordinate oral health management including the implementation of an integrated maternal and oral health policy. Community Dentistry specialists are trained to assess the dental needs of the population, conduct oral health surveys and surveillance of oral health and disease. Furthermore, they are trained to plan and implement appropriate evidence-based interventions, formulate oral health policies and strategies as well as to manage public oral health services (27).

**OBJECTIVE 2: Strengthen health system delivery platforms by addressing the World Health Organisation (WHO) “building blocks” for quality maternal and child oral health services along the continuum of healthcare**

This objective is based on the MCWH policy draft goal for mothers, which is to ensure access to high quality antenatal care (ANC) and quality care during and after delivery to mothers and their babies (33). An IMCOH policy would include children, adolescents and women of child-bearing age. Due to the high prevalence of HIV and AIDS in South Africa, particularly among mothers and children, education on common oral manifestations of HIV is essential for this vulnerable population. In addition, it would include addressing the myth that pregnant women should not seek oral health treatment during their pregnancy because it puts the foetus's health at risk.

In the draft MCWH policy, the goal for children is to enable each child to reach his/her maximum potential within the resources available, and to enable as many children as possible to reach adulthood with their potential uncompromised by illness, disability, environmental hazard or unhealthy lifestyle (35).

Oral health is the gateway to general health. If a child's oral health could be optimized from birth, their general health would only improve, making it easier for them to reach their full potential. Children can be accessed at the mother-to-child clinics that will ensure early intervention and referral. In general, the integration of health services aims to improve service efficiency and quality, which consequently maximises the use of resources and opportunities (11). In contrast to a vertical approach (focus on a single risk factor or intervention), an integrated approach consists of a collection of core interventions that can be considered part of a health care “package” (10). The common risk factor approach (CRFA) considers risk factors which may be common in the numerous chronic conditions in the context of the larger socio-environmental milieu. Diet, hygiene, smoking, alcohol use, stress, and trauma all have an impact on oral health. Since these causes are shared by a number of other chronic diseases, a collaborative (inter-professional) approach is preferable to a disease-specific approach (11). One of the principal objectives of the South African Oral Health Promotion Framework will be to incorporate oral health promotion activities into general health promotion using the CRFA. Importantly, the bidirectional relationship between oral health and other diseases and conditions provides compelling evidence for a bidirectional relationship between oral health and primary health care (11), especially to ensure the continuum of health care.

**OBJECTIVE 3: Promote coordinated, relevant, trans-multi-disciplinary and inter-sectoral community engagement to improve pregnancy and oral health outcomes**

Health care workers (HCW) are key role players for the implementation of the policy across the healthcare continuum. By encouraging the integration of medical, dental, and other health-care services, health care providers will be enabled to promote collaborative efforts with individuals, families, communities, policymakers, and governments, in order to offer easily accessible and consistent oral health messages and services to pregnant women, infants, and children, particularly those from vulnerable populations who are at increased risk for health disparities (47).

At primary health care facilities, HCW are the first point of contact for pregnant women, thus it is imperative that oral health services be included and an integral as part of a comprehensive health package. Health care workers should be trained to carry out oral health screening, oral health promotion and education and to refer if need be.

Inter-professional collaboration is a better way of providing comprehensive health care, and policymakers are leading the movement towards more integrated health care (10). There is a need for effective policies on interdisciplinary approaches to improve the oral health of underserved populations. Primary oral health care is not a new concept; in fact, it forms the basis of the United Nations Millennium Declaration Goal: “All people, everywhere shall have access to a skilled, motivated, and facilitated health worker within a robust health system” (51).

Promoting inter-professional education and collaborative practices to improve the quality and accessibility of care in underserved areas is necessary to combat the increasing prevalence of dental caries in South African children. Since infants and young children are more likely to attend post-natal and mother-to-child visits than seek preventive oral health care, cross-trained health (medical and nursing personnel) may be able to provide early oral health assessments and guidance through early primary-care visits (47).

Furthermore, in order to expand community access, increasing the scope of practice of community health workers (CHW) to include oral health promotion and education for mothers and children will be an efficient use of resources and benefit underserved communities. CHW can advocate for the importance of primary teeth to parents, caregivers and the community at large, and raise awareness of the negative impacts of childhood dental caries on quality of life of young children (48). In the West Rand District of Gauteng, CHWs are trained in basic oral health as they are inducted in the district in efforts to incorporate oral health into their activities. A basic oral health manual for CHWs has been developed for this purpose and tabled for national dissemination later. This is an imperative step toward incorporating oral health into the activities of CHW (52).

**OBJECTIVE 4: Establish a sustainable and contextual surveillance system for maternal and child oral health**

A key component of the policy process is policy evaluation. This would only be possible with a surveillance strategy that is built into a standardized and reliable oral health information system (40). Monitoring is commonly used to describe the process of systematically gathering data to provide answers to such inquiries. When the primary focus of an evaluation is to determine “how successfully a project, program, or policy is being implemented against predicted results,” the term performance monitoring is frequently used. The importance of monitoring is determined by key stakeholders' perceived need to learn more about what is going on the ground (53).

Ramphoma has suggested that oral health issues in South Africa have been ineffectively addressed mainly due to the absence of a systematic oral health surveillance strategy (27). Public health action relies largely on linking data from surveillance to health policies and programmes. Proper surveillance ensures that countries, and communities, have the necessary information for immediate disease control or to plan strategies towards disease prevention. Ultimately, surveillance aspires to assist governments as well as health authorities or professionals to control disease burdens affecting their populations (54). However, such information systems rely on the collection of contextually appropriate oral health indicators.

The information that an indicator provides must cover relevant public oral health disease burdens in a way that it is: measurable and robust, easy to understand, be relevant to oral health-related quality of life, clearly related to common modifiable risk factors and be instrumental to oral disease prevention, as well as, the promotion of oral health through health systems response (10). A minimum set of oral indicators at Provincial and District level needs to be established that can be tailored to target populations.

Monitoring of oral health services is critical for resource, people, and infrastructure planning (5). Regular audits will provide patient utilisation trends, types of dental care provided and the community disease burden. The type and frequency of dental treatments provided can be used to determine human resource management, infrastructure development, and equipment maintenance. It will also inform policies regarding personnel and dental resource distribution to achieve optimal health care delivery. Mukhari-Baloyi et al., suggest that the NOHP, which is currently the operational framework for oral health activities in South Africa, should be reviewed to identify flaws and improvements to inform a more appropriate and relevant to the South African context policy (2).

## Concluding remarks

South Africa's public health system, like many other public systems in the world, has a strong reciprocal obligation to provide the best possible care in order to protect its people, especially vulnerable children, from oral diseases such as dental caries. Long-term problems in South Africa include reducing inequities in income, health, and education, and creating possibilities for many more individuals to survive childhood, realize their full human potential, and live healthy, productive lives. Although, public policies should ensure that the democratic values of a country are respected and maintained, it is equally important that these policies are responsive to the challenges faced by the society (55).

In pursuit of universal health coverage, the recently released National Health Insurance (NHI) Bill aims to build on these reforms to improve quality, coverage, and equity, in response to South Africa's healthcare system's poor performance, persistent inequities, and subgroup vulnerability. Given the ongoing healthcare reforms, quantifying access to health-care services is critical (30). Improving access to sustainable and effective health care services is a top objective throughout the medium term. Short- term actions should consist of bolstering public health care services, enhancing resource allocation policies, and educating healthcare professionals (29). Integrated primary health care services have been observed to increase and support the performance of the health care system such as the NHI by creating supportive environments (56–59).

Despite the growing attention towards an integrated oral health and primary health care system, there is a paucity of literature on oral health integration models, and it remains unclear how and in what contexts this approach is being applied and is successful in practice. Even though there is widespread prevalence of childhood caries in South Africa, managing this condition in this vulnerable population is currently not a priority. Any policies and programmes which emphasise the integration of oral health in existing primary health care should be supported. Inter-professional collaboration, through the training and participation of general health care professionals and CHWs, to provide oral health care for children is essential to eliminate inequities in child oral health.

The integration of oral health care into PHCs, especially maternal and child services, by establishing interdisciplinary networks, training non-dental care providers, modelling oral health champions, enabling care linkages and care coordinated processes, and utilising e-health technologies has the potential to improve the oral health of the South African under six population. Furthermore, more scientific, evidence-based, and rigorous evaluation research is required to provide data on the cost- effectiveness and long-term outcomes of oral health integrated models. The strength of this review is that it seeks to include the current contextual body of knowledge on the topic and may offer new perspectives for further research work. Although informally discussed, integrated maternal and child oral health care has not been formalized into actionable plans. Thus, this proposed policy sheds light into the South African context. The limitations are that proper surveillance data could not be cited to inform decision making due to the lack of oral health information systems in South Africa. Additionally, a specific framework was not proposed but this could be looked into in the near future.
